# BattleSound: A Game Sound Benchmark for the Sound-Specific Feedback Generation in a Battle Game

**DOI:** 10.3390/s23020770

**Published:** 2023-01-10

**Authors:** Sungho Shin, Seongju Lee, Changhyun Jun, Kyoobin Lee

**Affiliations:** School of Integrated Technology, Gwangju Institute of Science and Technology, Gwangju 61005, Republic of Korea

**Keywords:** deep learning, sound event detection, haptic feedback, voice chat activity detection

## Abstract

A haptic sensor coupled to a gamepad or headset is frequently used to enhance the sense of immersion for game players. However, providing haptic feedback for appropriate sound effects involves specialized audio engineering techniques to identify target sounds that vary according to the game. We propose a deep learning-based method for sound event detection (SED) to determine the optimal timing of haptic feedback in extremely noisy environments. To accomplish this, we introduce the *BattleSound* dataset, which contains a large volume of game sound recordings of game effects and other distracting sounds, including voice chats from a *PlayerUnknown’s Battlegrounds* (PUBG) game. Given the highly noisy and distracting nature of war-game environments, we set the annotation interval to 0.5 s, which is significantly shorter than the existing benchmarks for SED, to increase the likelihood that the annotated label contains sound from a single source. As a baseline, we adopt mobile-sized deep learning models to perform two tasks: weapon sound event detection (WSED) and voice chat activity detection (VCAD). The accuracy of the models trained on *BattleSound* was greater than 90% for both tasks; thus, *BattleSound* enables real-time game sound recognition in noisy environments via deep learning. In addition, we demonstrated that performance degraded significantly when the annotation interval was greater than 0.5 s, indicating that the *BattleSound* with short annotation intervals is advantageous for SED applications that demand real-time inferences.

## 1. Introduction

Sound is a very common and straightforward method for increasing the level of immersion in games [1]. In recent times, haptic sensors coupled to a gamepad or headset have been frequently utilized for game feedback to deliver sound as physically represented feelings [2,3]. However, providing haptic feedback for appropriate sound effects requires specialized audio engineering techniques to detect target sounds in real-time. Furthermore, numerous considerations, such as when to deliver feedback and eliminate distracting noise-like sounds, must be made [4]. The most straightforward approach for recognizing target game effects is to obtain the signal directly from the game engine. Major video game manufacturers, including Sony and Microsoft, integrate their gamepad software into their PlayStations, enabling direct communication with game engines. When a predetermined signal is received, haptic sensors linked to the gamepad provide feedback. However, this strategy cannot be applied to non-negotiated games. Another approach is to filter the target sound using acoustic characteristics, such as frequency and volume [5]. You et al. introduced sound-specific vibration interfaces that provide haptic feedback in response to low-frequency and loud sounds (e.g., gunshot) [6]. Similarly, Lee et al. pioneered haptic interfaces for mobile environments, which convert low-pass filtered sound into vibrations for an interactive and realistic physical sensation [7]. Although filter-based methods enable the integration of haptic devices into a previously unconsidered variety of games, they are not as precise as direct communication. In addition, game sounds are mixed with many sound effects from different sources; as a result, simply filtering the target sounds by frequency or volume is insufficient. For instance, when a low-pass or volume filter is employed to filter the game sound, a loud voice chat may often be confused with gun sounds [5].

**Figure 1:**
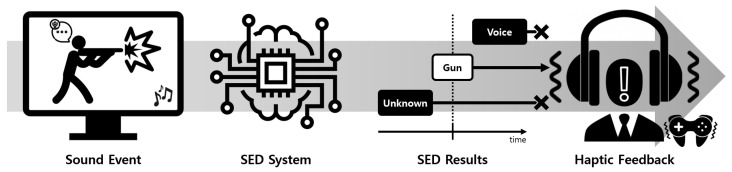
Example application of the *BattleSound* to automatic sound-specific haptic feedback generation by detecting a target weapon sound and a filtering distracting sound, such as voice chat.

To address the aforementioned issues, we propose using deep learning to detect target sounds in video games. By training on a large dataset of game sounds, deep learning can accurately detect specific sounds in noisy environments while still maintaining generalizability (Figure 1). Sound event detection (SED) is a technique for identifying and extracting specific sounds from large volumes of audio input [8,9,10]. We believe that, with the appropriate dataset, deep learning can be used for SED to detect game effects such as gunshots and explosions. Additionally, voice activity detection (VAD) can be used to filter out voice chat, which is often misclassified as target sounds. VAD involves detecting voice from the audio input, and deep learning algorithms such as long short-term memory (LSTM) have been shown to have high success rates for this task [11,12,13]. Both SED and VAD techniques involve training deep neural networks using large annotated audio datasets that are specialized for the target domain.

The construction of large audio benchmarks has facilitated the development of deep learning methods for sound detection, such as the *AudioSet* dataset, which contains over two million human-labeled 10-s sound clips and 632 audio event classes [14]. However, many of these benchmarks are limited to simple applications, such as environmental and bird sound classification, and are not representative of real-world scenarios, such as noisy video game environments [15]. There is a need for sound benchmarks that accurately represent these environments. To fully exploit the advantages of deep learning for game sound detection, we constructed the *BattleSound* dataset, which contains a large number of game audio clips and annotations of game effects. Each audio clip was collected from the *PlayerUnknown’s Battlegrounds* (PUBG) game [16], a battle royale game that features a variety of sound effects, including weapons, vehicles, footsteps, and voice chat. We annotated the audio clips into three categories: WEAPON, VOICE, and MIXTURE. Samples labeled as WEAPON and MIXTURE were utilized for weapon sound event detection (WSED) and samples labeled as VOICE and MIXTURE were utilized for voice chat activity detection (VCAD). We developed baseline models for the WSED and VCAD tasks using mobile-sized deep learning models [17,18]. This was to demonstrate that haptic feedback can be generated when a weapon sound is detected via WSED, whereas VCAD detects voice to avoid misclassification as a weapon sound, which results in unwanted haptic feedback.

**Figure 2:**
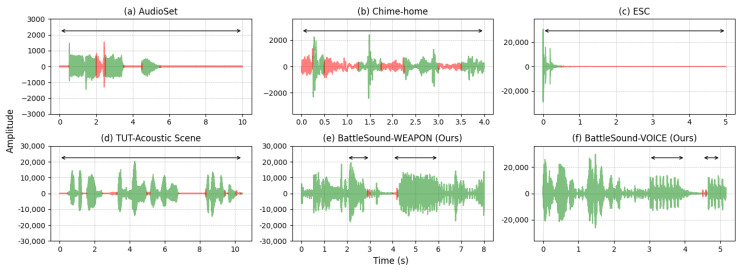
Visualization of the randomly selected sample from the audio benchmark dataset. The black arrow indicates the region annotated as target sound; the red colored region indicates mislabeled parts such as non-target sound annotated as target or vice versa.

The primary characteristics that differentiate WSED and VCAD from SED and VAD are intra-class variations and noisy environments. The PUBG game features a diverse range of user voices representing a variety of nationalities, genders, ages, weapons, and other distracting sounds. Owing to these variations within classes, it is difficult for deep learning models to learn the optimal hyperplane for classifying inter-class samples. PUBG sets approximately 100 players simultaneously against each other until a player or team remains. This indicates that numerous audio clips contain a mixture of sounds from multiple sources, which complicates the task of identifying the target sounds. Furthermore, because both tasks must be completed concurrently with game play, deep learning on a mobile device must make real-time inferences from short audio clips. However, as described in Table 1, existing benchmarks [14,19,20,21,22,23,24,25,26,27,28] contain audio clips annotated with a large interval (i.e., low-resolution). Hence, numerous samples contain mislabeled frames that do not correspond to the correct labels, as illustrated in Figure 2. Previous studies refer to this as the weak label problem [11,12,13,29,30]. Yu et al. asserted that large portions of the VAD dataset contained incorrectly labeled frames, resulting in a degradation of VAD performance [11]. Cho et al. [12] and Ghosh et al. [13] also criticized the lack of correctly labeled frames in massive datasets and proposed recurrent-based architectures to deal with incorrectly labeled frames by using sequential information. Similarly, we demonstrate that incorrectly labeled frames confuse the sound classification network; hence, a strongly labeled dataset is required for an accurate model, especially in real-time applications and noisy environments.

In the construction of *BattleSound*, we focused on the interval between annotations to improve the sound detection performance in noisy game situations. It is necessary to annotate a large number of audio clips with a short interval (i.e., high-resolution), which is referred to as a strong label. However, there are no concrete rules to define the criterion for a strong label. If the interval between annotations is too short, the target sound may not be included; if it is too long, confused frames may be included, reducing the reliability of the label. Salamon et al. [24], Foster et al. [21], and Kumar et al. [29] argued that a chunk size of 4 s is the optimal balance of the trade-off between the temporal resolution of annotation and human labor. However, based on this study, we discovered that a 4 s interval between annotations is insufficient for real-time WSED and VCAD tasks. Given that annotation tasks are performed by humans, the human auditory system must be considered. Rosen et al. discovered that although auditory information occurs at 20–40 millisecond (ms) intervals, syllabicity and prosodic phenomena occur at 100–200 ms intervals or longer [32]. Furthermore, homologs in the left hemisphere preferentially extract information from short temporal integration windows (20–40 ms), whereas homologs in the right hemisphere preferentially extract information from long integration windows (150–250 ms) [33]. Consequently, we established a 500-ms threshold for a strong label, which enables the human annotator to make a judgment based on approximately two syllables of sound.

Finally, we assess the performance of various deep learning models on *BattleSound* and emphasize the benefits of *BattleSound*’s short annotation intervals for real-time game sound event detection. Our main contributions can be summarized as follows:

- We proposed a significant amount of high-resolution game sound benchmarks, *BattleSound*, which includes a variety of different voice, gun, and explosion sounds.
- Using deep learning models trained on *BattleSound*, voice chat and weapon sounds can be recognized in real-time, enabling automated processes for sound-specific feedback production.
- Based on the human auditory system, we established the 0.5 s criterion for a strong label and demonstrated that strongly labeled audio data can significantly improve performance in real-time WSED and VCAD.

The remainder of this paper is organized as follows. In Section 2, previous works related to constructing large-scale game sound benchmarks were described. In Section 3, we describe the pipeline used to construct the *BattleSound* and its characteristics. Section 4 and Section 5 present experimental results assessing the performance of deep learning models on *BattleSound* and discuss the findings. Finally, in Section 6, we provide a summary of this study.

## 2. Related Works

### 2.1. Audio Dataset

The development of deep learning methods for sound detection is facilitated by the construction of massive audio benchmarks [14,19,20,21,22,23,24,25,26,27,28,31,34]. Gemmeke et al. created an efficient pipeline for generating massive audio datasets from YouTube and provided *AudioSet*, which contains 632 audio event classes and 2,084,320 human-labeled 10 s sound clips [14]. As *ImageNet* [35] contributed significantly to the development of image-based deep learning models [36,37,38], *AudioSet* contributed significantly to the development of audio-based deep learning models [39,40,41,42]. The *UrbanSound* [24] dataset contains 27 h of urban environmental sounds, the *SINS* dataset [23] contains 200 h of environmental sound, and the *LibriSpeech* [27] dataset contains 1000 h of speech corpus (Table 1). Although these datasets contain sufficient audio data, they are limited to simple applications (e.g., environmental and bird sound classifications), which are not applicable to real-world scenarios [15]; hence, there is a dearth of sound benchmarks that accurately represent noisy game environments.

### 2.2. Sound Event Detection (SED)

Sound event detection (SED) is the task of identifying and classifying specific sounds within an audio signal. It is a challenging problem due to the wide variety of sounds that can occur in real-world environments and the presence of noise and other interfering factors. SED has numerous applications, including sound-based monitoring and surveillance, audio content analysis, and audio-visual scene analysis.

There are various approaches to SED, including traditional methods [5,6,7] that rely on hand-crafted features and machine learning algorithms and more recent deep learning-based approaches [8,9,10,43,44]. Traditional methods often extract features, such as spectral and temporal information, from the audio signal and use these features as input to a classifier such as a support vector machine or decision tree. Deep learning-based approaches, on the other hand, typically involve training a neural network on large amounts of annotated audio data and using the trained network to classify audio signals. SED has been applied in various fields, such as identifying bearing faults in noisy industrial environments using deep neural networks [8], recognizing the cow sound [45,46], and detecting respiratory diseases in patients’ voices [9,10]. More recently, advanced methods such as self-supervised learning [43,44], which uses large amounts of unlabeled audio data for pre-training a model, and multi-task learning [47], which involves a joint learning approach for sound event detection and localization, have been utilized to improve SED performance.

### 2.3. Voice Activity Detection (VAD)

Voice activity detection (VAD) is the task of identifying periods of speech or non-speech in an audio signal. It is a common pre-processing step in many speech-processing systems, as it can help to separate speech segments from noise and other interfering sounds.

Cho et al. [12] and Ghosh et al. [13] analyzed the characteristics of voice that distinguishes it from other sounds and filtered the voice using pre-calculated features. Yu et al. utilized a deep learning algorithm known as long short-term memory to improve the performance of the VAD task by training the network to extract meaningful voice features from large audio samples [11]. In recent years, self-supervised learning techniques have also been applied to VAD, using large amounts of unlabeled audio data for pre-training the model [48]. These approaches have shown promising results for VAD, particularly in noisy and reverberant environments.

## 3. BattleSound Dataset

PUBG is a battle royale game in which 100 people compete on an island with a variety of weapons and strategies. Owing to the absence of strict constraints, each player has a wide range of options in the game. For instance, a player can actively combat enemies with a bomb and a rifle, whereas another can drive a car, boat, and tank toward strategic areas. We constructed the *BattleSound* by aggregating numerous types of sounds from PUBG and categorizing them as VOICE, WEAPON, and MIXTURE. VOICE denotes the voice recorded from Team Voice, which is a communication tool for the team members; WEAPON denotes the effect sound of weapons such as guns and bombs; and MIXTURE refers to the combined sound of VOICE and WEAPON. Certain voice conversations and weapon sounds may overlap during battles, resulting in distinct representations that make it challenging to identify a single voice or weapon sound.

### 3.1. Dataset Characteristics

Table 2 summarizes the basic statistics of *BattleSound*. A total of 25,367 samples with a duration of 3.52 h can be used as target samples (VOICE and MIXTURE) for voice chat detection. Similarly, 26,142 samples with a total duration of 3.62 h can be used as target samples (WEAPON and MIXTURE) for the weapon sound detection. *BattleSound* contains a sufficient number of samples for the real-time sound event detection using deep learning.

**Figure 3:**
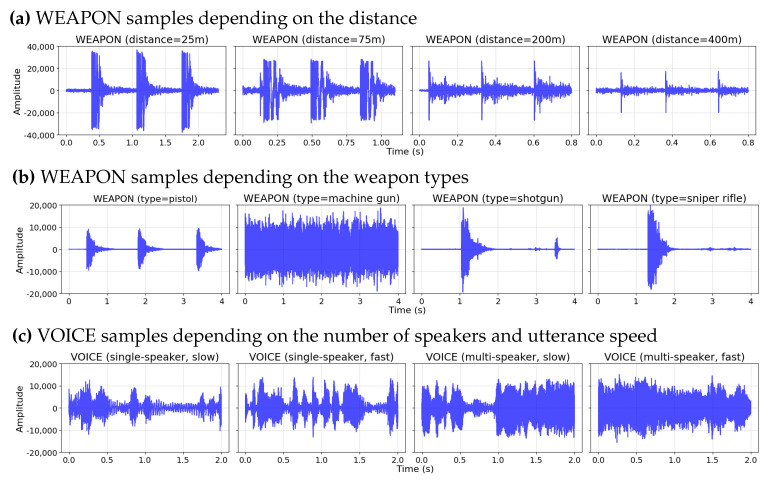
Visualization of the variations in *BattleSound* samples. (**a**,**b**) show the variation in the WEAPON samples depending on the distance and weapon types, respectively. (**c**) shows the variation in VOICE samples depending on the number of speakers and their utterance speed.

Figure 3 depicts the various types of audio signals included in *BattleSound*. During the battle, players shoot the gun from a variety of angles and distances. Figure 3a illustrates the variation in sound signal produced when the same type of gun is fired at distances ranging from 25 to 400 m. Figure 3b depicts the variation in gun types observed when players shoot a variety of different types of guns at the same distance. Within the 4 s clips, shotguns and sniper rifles are fired only once; however, machine guns can be fired nearly 20 to 40 times within a brief interval. Owing to the interaction of several conditions (e.g., distance, angle, and gun type), WEAPON samples exhibit a high degree of variation. Similarly, VOICE samples exhibit a high degree of variation, as multiple players speak concurrently and each player produces a unique sound with a unique utterance speed. As players communicate with their teams (2–4 players) via voice chat, some VOICE clips include a single speaker, whereas others feature several speakers with overlapping sounds, as depicted in Figure 3c.

### 3.2. Data Collection

**Figure 4:**
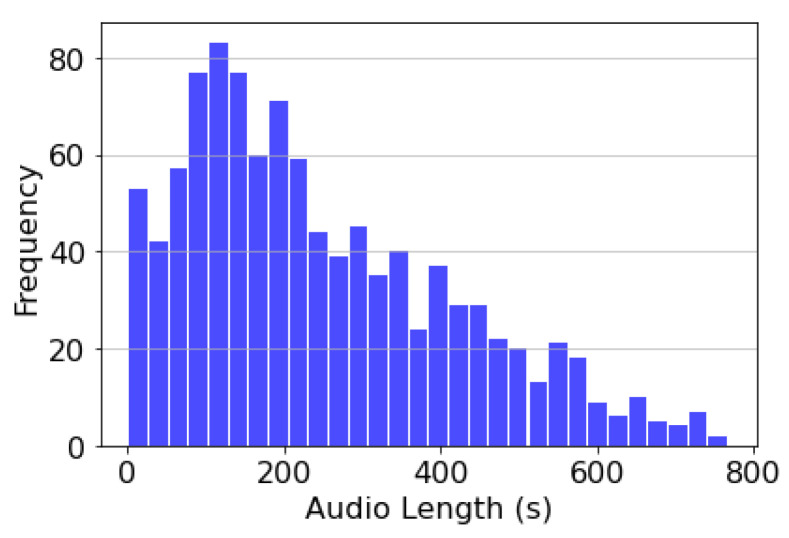
Histogram of the audio clips’ length included in the *BattleSound*.

The *BattleSound* is a collection of audio files from PUBG that have been crawled from YouTube. We collected YouTube video clips containing the keywords “PUBG” or “battle ground” in the title in both English and Korean. In crawling the clips, we used two distinct strategies to remove irrelevant files prior to human annotation. First, only files with a duration of less than 16 min were considered candidates for human annotations. Because the average duration of the PUBG was 17 min, files longer than 17 min were more likely to contain sounds that were not from the PUBG. Second, three human annotators pre-screened crawled videos to ensure that they contained only PUBG-related content and not distracting elements, such as advertisements and TV show clips. Subsequently, a total of 1038 video clips (55-h) were selected, and each clip was segmented (n = 513,006) into clips with a 0.5 s interval for the following human annotations. All files were downloaded at a sampling rate of 48 kHz and subsequently downsampled to 16 kHz using the *AudioSet* protocol [14]. As illustrated in Figure 4, *BattleSound* contains clips ranging in length from a few seconds to 15 min.

### 3.3. Human Annotation

Almost all segments contain a single sound when crawled files were segmented at a 0.5 s interval, which is a significantly higher resolution than the existing benchmark (Table 1). Thirty-six annotators manually listened to and labeled entire segments every 0.5 s using our labeling software. The software was designed to run entirely on a keyboard, allowing annotators to quickly and easily label large numbers of audio samples. Audio files were segmented and played consecutively using a predefined interval (0.5 s in our studies). When a single audio segment was played, annotators were required to press a key that indicated the presence of a label. By repeating the aforementioned procedure for each clip, all audio files were annotated, and the resulting files were saved as a NumPy array. Labeling 10 s audio clips with a 0.5 s interval required an average of 15 s. Our labeling software, along with the *BattleSound* dataset, code, and demo videos, can be downloaded from our project page (https://sites.google.com/view/battlesound (accessed on 5 January 2023)).

### 3.4. Validation and Quality Assessment

Owing to the uncertainty associated with annotations, we validated them twice to ensure accurate labeling. First, 36 annotators validated the entire segment to ensure that each label corresponded to a specific piece of audio content. Second, four artificial intelligence researchers with a thorough understanding of this project scrutinized all annotation files and corrected mislabeled and unlabeled cases. Six and half hours of clean target segments (VOICE, WEAPON, and MIXTURE) were constructed after the validation process (Table 2). The constructed dataset exhibited 100% quality from 100 randomly selected segments for each category, as determined by the *AudioSet* quality assessment process [14].

### 3.5. Audio Data Representation

For each *BattleSound* clip, we provided three different audio representations that are common in the SED and VAD. The following is a detailed description of the procedures used to convert an audio signal into other representations.

1. **Audio Signal** is the simplest form of sound and consists of a 1D waveform array, which indicates the amplitude of sound in each time stamp. All the samples in the *BattleSound* has a 16-kHz sampling rate. Training and validation for each sample ($x_{signal}$) was 0.5 s, and $x_{signal}$ has a dimension of 1 × 8000.
2. **Spectrogram** is a visual representation of sound created by applying short-time Fourier transformation (STFT) to the audio signal. STFT is a Fourier-related transform used to estimate the sinusoidal frequency and phase content of particular parts of the changing signal.
  (1) $$STFT\left(x\right)=\sum_{n=0}^{N-1}x\left[n\right]\cdot W\left[t\right]$$
  (2) $$x_{spec}=STFT\left(x_{signal}\right)$$
  $x_{signal}$ and $x_{spec}$ denote the 1D audio signal and 2D spectrogram, respectively. x[n] and window function ($W\left[t\right]=e^{\left(-j2\pi fn\right)}$) denote a part of raw signal and window function, respectively. Those are utilized to calculate the spectrum, where *t* and *n* indicate the time and frequency bins, respectively. We utilized the STFT parameters of a 500 ms window length, 25 ms hop length, and 512 bins. The converted spectrogram ($x_{spec}$) of the single sample had dimensions of 1 × 257 × 41.
3. **Mel-spectrogram** is generated by applying mel-filter to the spectrogram. Humans perceive frequencies more sensitively to a lower frequency range than a linear scale. By imitating the nonlinear characteristics, a mel-filter ($H_{m}\left(k\right)$) was proposed for the human-like representation of sound.
  (3) $$H_{m}\left(k\right)=\left\{\begin{array}{cc} 0 & k<f(m-1)\\ \frac{k-f(m-1)}{f(m)-f(m-1)} & f(m-1)<k<f(m)\\ 1 & k=f(m)\\ \frac{f(m+1)-k}{f(m+1)-f(m)} & f(m)<k<f(m+1)\\ 0 & k>f(m+1) \end{array}\right.$$
  Mel-filter banks produced by the function of mel-filters ($H_{m}\left(k\right)$) were used to extract frequency domain features. Here, f(m) is the Hertz function calculated from the mel (*m*), and the mel-filter banks are collections of mel-filters for different *k*. Filters are densely situated in the low-frequency region compared to the high-frequency regions to emphasize the differences in the low-frequency region.
  (4) $$x_{mel}=H_{m}\left(k\right)*x_{spec}$$
  The mel-spectrogram was generated by passing the input spectrogram into the mel-filters calculated using Equation (4). Here, $x_{mel}$ indicates mel-spectrogram. In this study, we utilized mel-filters with 41 mel-coefficients to the frequencies ranging between 300 and 8000 Hz after conversion into the spectrogram. The dimension of the converted mel-spectrogram ($x_{mel}$) was 1 × 41 × 41.

## 4. Experiments

### 4.1. Task Definition

*BattleSound* contains a large amount of audio content recorded from game environments with a 0.5 s annotation interval. To fully exploit the advantages of *BattleSound*, we developed baseline models for the following two tasks: real-time voice chat activity detection (VCAD) and weapon sound event detection (WSED). For both tasks, 80% of the samples were utilized for training, and the remaining was used for validation.

**Real-time voice chat activity detection (VCAD)** is a task that identifies voices in the streamed audio input. Typically, two to four players forming a team can communicate via voice chat while playing PUBG. Because multiple players speak concurrently and loudly, several parts of the recorded voice contain noise and overlapping sounds. In addition, weapon sounds are frequently mingled with voices, making them difficult to distinguish. To recognize the voice in the streamed audio input, we developed a VCAD model using deep learning. The VOICE-and MIXTURE-labeled samples in the *BattleSound* were considered target voice samples, whereas the samples labeled with WEAPON were used as non-target samples.

**Weapon sound event detection (WSED)** is a task that entails real-time detection of weapon sounds, such as gun and bomb from streamed audio input. For a realistic feeling, numerous game devices provide visual or haptic feedback in response to game effects, such as shooting or striking. If the game’s effects can be detected via sound, the timing of the feedback delivery can be determined automatically. Therefore, we developed a deep learning model capable of detecting weapon sounds from streamed audio. We used the WEAPON-and MIXTURE-labeled samples in *BattleSound* as target samples and the VOICE-labeled samples as non-target samples.

### 4.2. Baseline Methods

Convolutional neural networks (CNN) have been widely applied to a variety of classification tasks, including 1D signal analysis [11,49,50] and 2D imaging analysis [51,52,53]. CNN has the advantages of extracting spatial information from the inputs by sliding the fixed size of kernels with trainable weights. For the time-series input, such as audio signal, understanding the temporal features is also important for the classification; recurrent neural network (RNN) and long short-term memory (LSTM) have widely been utilized to extract the temporal features from the time-series input and perform the classification using those features. In this study, we established baseline models using CNN [17] and CRNN [18], which combines the LSTM model after the CNN model, and utilized the two different representations of sounds as input (1D audio signal and 2D mel-spectrogram image).

Because our tasks should work in real-time, mobile-sized CNN [17] and CRNN [18] were utilized. For all models, only three convolutional blocks were utilized, as suggested by Sehgal et al. [17]. These models are comparable with the edge devices because of the small number of parameters: 27,542 for 1D CNN, 394,870 for 1D CRNN, 373,122 for 2D CNN, and 220,802 for 2D CRNN. In real-time applications, all models classify the audio input every 25 ms using the recently streamed sound of 0.5 s. The detailed structures and parameters are presented in Table 3 and Table 4.

### 4.3. Label Resolution Adjustment

*BattleSound* includes high-resolution annotations on all samples to ensure superior performance in real-time applications. To assess the efficacy of high-resolution labels, we downsampled the labeling resolution from 0.5 to 2, 4, and 8 s and compared baseline performance across resolutions. To perform the downsampling, we used a sliding window with the length of the target resolution, which can be slid without overlapping. If a window contains at least one target sound, all frames contained within the window are labeled with the target sound. It is expected that the mislabeled frames included in the window will degrade the performance of the baseline tasks, particularly in real-time applications. To ensure a fair comparison, only the training samples were downsampled, whereas the validation samples were strongly labeled (0.5 s interval). To compare the performance based on label resolution, we trained a 2D CNN model with mel-spectrogram inputs of equal length to the resolution; for the evaluation, validation samples of length 0.5 s are repeated in order to match the length of input samples to train samples.

## 5. Results

### 5.1. Weapon Sound Event Detection Results

**Figure 5:**
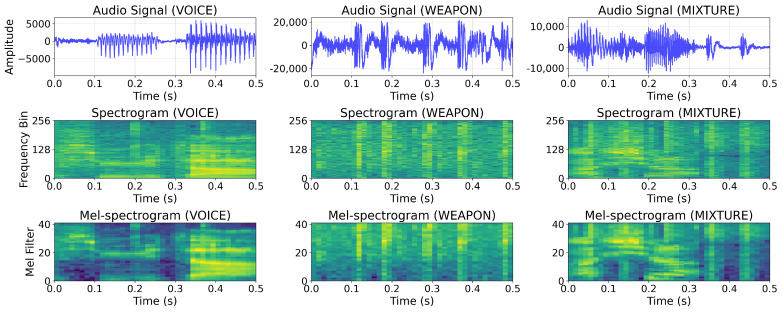
Samples of the *BattleSound*. We presented three different types of sound representations: audio signal, spectrogram, and mel-spectrogram. The VOICE sample exhibited highly activated regions primarily in the low-frequency domain, as depicted in the first column, whereas the WEAPON sample had highly activated regions through the frequency domain, as depicted in the second column. The differences between the VOICE and WEAPON samples in the low-frequency domain were well captured in the mel-spectrogram.

**Figure 6:**
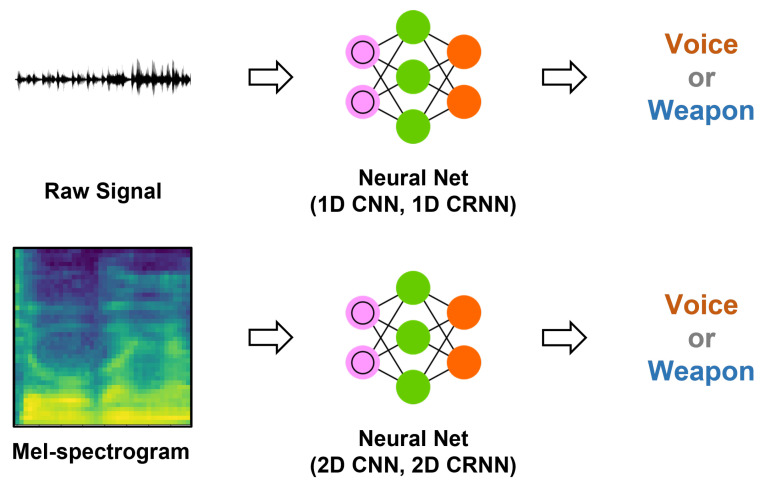
Architectures of deep learning models which use the raw signal and mel-spectrogram as input, respectively. They are used to detect voice or weapon sounds.

As a baseline, we compared the WSED performance of CNN and CRNN models for two distinct representations (1D audio signal and 2D mel-spectrogram image). In general, weapon sounds and voice exhibit distinct patterns in the frequency domain (Figure 5 and Figure 6); highly activated regions exist throughout the frequency domain of the weapon sound, whereas voice samples exhibited highly activated regions primarily in the low-frequency domain; the mel-spectrogram highlighted the frequency domain features, indicating that the 2D models outperformed the 1D models. In addition, the accuracy of the CNN models exceeded that of the CRNN models by 0.6% for 1D signals and 0.5% for 2D mel-spectrogram images in the Table 5. Typically, weapon sounds exhibit short-duration, repetitive patterns; hence, the CNN model, which has an advantage in capturing local spatial patterns, can demonstrate superior performance even more so than the CRNN. The CNN model that inputs the 2D mel-spectrogram images attained an average accuracy of 96.02% and enabled mobile-sized deep learning models to detect weapon sounds from the streamed audio inputs in real-time.

### 5.2. Voice Chat Activity Detection Results

**Figure 7:**
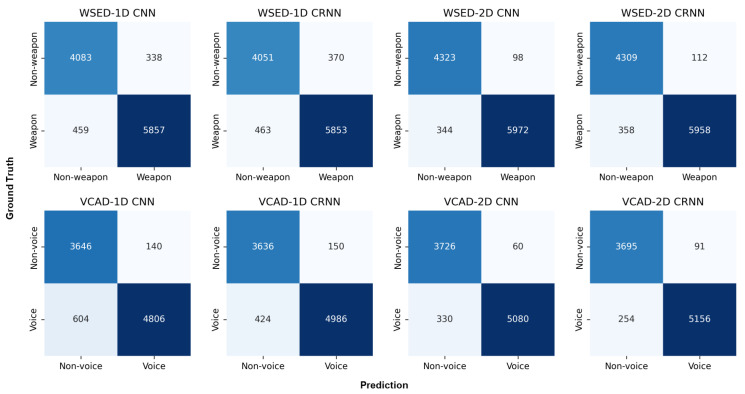
Confusion matrices for the WSED (top row) and VCAD (bottom row) tasks for four different models: 1D CNN, 1D CRNN, 2D CNN, and 2D CRNN. For each matrix, the rows represent the ground truth labels, and the columns represent the predicted labels. Dark blue represents the high scores.

We assessed the VCAD performance of the CNN and CRNN models for two distinct representations as a baseline. In addition, we evaluate the filter-based method on the VCAD task to highlight the efficacy of deep learning models. The energy filter [5] is a frequently used statistical model for VAD. It calculates the representative energy levels of voices and filters out other sounds with a lower energy level than the voice. However, this method frequently confuses the weapon samples of *BattleSound* with the voice. This is because weapon samples commonly have high energy levels owing to their high decibels in low-frequency regions; as a result, deep learning models trained on *BattleSound* significantly outperformed the energy filter [5] (64.86%). Similar to the WSED problem, 2D models performed better than 1D models in the VCAD task because mel-spectrogram images well represent the frequency domain’s properties. In contrast to the WSED task, the CRNN model outperformed the CNN model for both representations; because speech sounds have a long duration, the CRNN models that can capture both spatial and temporal features beat the CNN models that just concentrated on capturing local features. The CRNN model with 2D mel-spectrogram inputs attained an average accuracy of 96.37% and enabled mobile-sized deep learning models to detect voice from streamed audio inputs in real-time. We attached the confusion matrices for WSED and VCAD in the Figure 7.

### 5.3. Multi-Class Voice and Weapon Sound Event Detection

In our dataset, we have previously proposed two tasks: VCAD and WSED, which are formulated as two-way classification tasks, classifying sounds into target and non-target labels. However, it is also possible to evaluate a model on all available classes in the dataset in order to classify VOICE, WEAPON, and MIXED sounds using a single model. In Table 6, we present the results of this multi-class classification task. Similar to the two-way classification settings (VCAD and WSED), we find that 2D models perform better than 1D models, likely due to the well-represented features of mel-spectrogram images compared to 1D signals. The CNN model with 2D mel-spectrogram inputs achieved an average accuracy of 94.25%, demonstrating the ability of mobile-sized deep learning models to detect voice and weapon sounds simultaneously from streamed audio inputs in real-time.

### 5.4. Effects of Label Resolution on Sound Detection Performances

**Figure 8:**
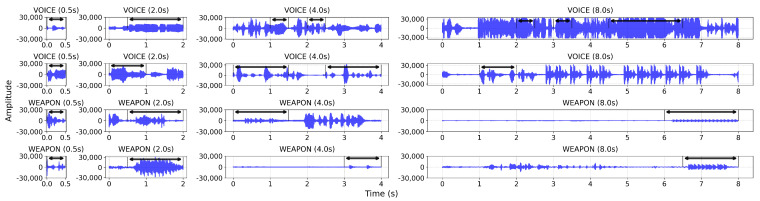
Audio signal samples depending on the label resolution. The black arrow denotes the frames identified as the target sound in the high-resolution labels. The parts omitted by the black arrow are non-target frames, often known as confused frames, which were incorrectly included due to the low-resolution labeling. As the resolution declines, a greater proportion of confused frames are present.

**Figure 9:**
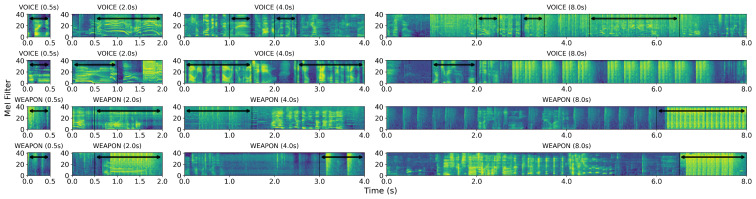
Mel-spectrogram samples depending on the label resolution. The black arrow denotes the frames identified as target sounds in the high-resolution labels. The parts omitted by the black arrow are non-target frames, often known as confused frames, which were incorrectly included due to the low-resolution labeling. As the resolution declines, a greater proportion of confused frames are present.

**Figure 10:**
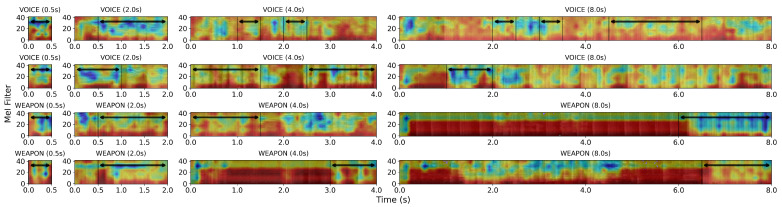
GradCAM results depending on the label resolution. Figure 9’s mel-spectrogram samples were utilized for the GradCAM analysis. Red-colored regions represent highly activated features, whereas blue-colored parts represent less active features. The black arrow denotes the frames identified as target sound in the high-resolution labels.

In Table 5, we demonstrated that our *BattleSound* dataset, with high-resolution labeling, enables mobile-sized deep learning models to detect target sounds in real-time. When we visualized the *BattleSound* samples with the different resolutions (2, 4, and 8 s), we found that a significant proportion of the samples were non-target sounds (Figure 8 and Figure 9). These non-target frames, also known as confused frames [11], can make the supervised learning process more difficult. To analyze the effects of confused frames on the supervised learning process, we used GradCAM [54], a visualization technique that identifies the parts of an input image that are most important for a given prediction made by a CNN. GradCAM results of 2D CNN models trained with low-resolution labels showed that the model not only concentrates on the target frames but also on the confused frames (colored red in Figure 10). This effect becomes more pronounced as the resolution degrades; as a result, for the WSED task, the 2D CNN model accuracy declined by 21.8% when the resolution degraded from 0.5 to 8 s, and for the VCAD task, the 2D CNN model accuracy degraded by 24.6% under the same conditions (see Table 7). These results suggest that high-resolution labeling is beneficial for learning the distinguishing characteristics for sound detection tasks that require real-time inferences.

## 6. Conclusions

We introduced the *BattleSound* dataset, which contains a large volume of high-resolution voice and weapon sounds. Using the *BattleSound*, we developed deep learning models for voice chat activity detection (VCAD) and weapon sound event detection (WSED) and achieved high performance in identifying target sounds, thereby enabling the generation of real-time sound-specific feedback. Furthermore, we demonstrated that annotation intervals are crucial factors affecting sound detection performance, particularly in noisy environments. The *BattleSound* is the first high-resolution game sound benchmark that focuses on sound-specific feedback generation by detecting the target sound via WSED and filtering the distracting sound via VCAD. Our study establishes a foundation for constructing a game sound dataset with high-resolution labeling and deep learning models for sound-specific feedback generation that can be widely applied to other game industries.

## Figures and Tables

**Table 1:** Description of the existing audio benchmark dataset. SED, ESC, and VCAD denote the sound event detection, environmental sound classification, and voice chat activity detection.

Dataset	Total Length (Hours)	Annotation Interval (Seconds)	Number of Classes	Task
*AudioSet* [14]	5790	10	632	SED
*Freefield1010* [19]	20	10	2	SED
*ToyADMOS* [20]	180	10	3	SED
*Chime-home* [21]	6.5	4	7	SED
*GunShot* [31]	50	2	2	SED
*URBAN-SED* [22]	28	10	9	ESC
*SINS* [23]	200	10	9	ESC
*UrbanSound* [24]	27	4	10	ESC
*ESC* [25]	17	5	10	ESC
*TUT Acoustic Scene* [26]	24	10	10	ESC
*BattleSound* (Ours)	6.7	0.5	3	WSED and VCAD

**Table 2:** Basic statistics of the *BattleSound*.

	VOICE	WEAPON	MIXTURE
count	21,904	22,679	3463
length	3.05 h	3.14 h	0.48 h

**Table 3:** Model specification of 1D and 2D CNNs. *k*: kernel size, *s*: stride, *p*: padding size, BN: batch normalization, out_dim: output dimension.

(**a**) The structure of 1D CNN, which inputs the audio signal		
**Layer Name**	**Composition**	**Output Size**
Input	-	1 × 8000
Conv1	10 Conv1D(*k* = 25, *s* = 3, *p* = 12) + BN + ReLU	10 × 2667
Max-pool1	MaxPool1D(*k* = 2)	10 × 1333
Conv2	20 Conv1D(*k* = 25, *s* = 3, *p* = 14) + BN + ReLU	20 × 446
Max-pool2	MaxPool1D(*k* = 2)	20 × 223
Conv3	40 Conv1D(*k* = 25, *s* = 3, *p* = 16) + BN + ReLU	40 × 78
Max-pool3	MaxPool1D(*k* = 3)	40 × 26
Flatten	Flatten	1040
Linear	FC(*out_dim* = 2)	2
(**b**) The structure of 2D CNN, which inputs the mel-spectogram image		
**Layer Name**	**Composition**	**Output Size**
Input	-	1 × 41 × 41
Conv1	10 Conv2D(*k* = 5, *s* = 2, *p* = 2) + BN + ReLU	10 × 21 × 21
Conv2	20 Conv2D(*k* = 5, *s* = 2, *p* = 2) + BN + ReLU	20 × 11 × 11
Conv3	40 Conv2D(*k* = 5, *s* = 2, *p* = 2) + BN + ReLU	40 × 6 × 6
Flatten	Flatten	1440
Linear	FC(*out_dim* = 256)	256
Linear	FC(*out_dim* = 2)	2

**Table 4:** Model specifications of 1D and 2D CRNNs. *k*: kernel size, *s*: stride, *p*: padding size, BN: batch normalization, out_dim: output dimension. Since LSTM model’s inputs are time-series data (channel×time), frequency domain parts of the mel-spectrogram are viewed as a channel dimension in order to match time-series forms.

(**a**) The structure of 1D CRNN, which inputs the audio signal		
**Layer Name**	**Composition**	**Output Size**
Input	-	1 × 8000
Conv1	64 Conv1D(*k* = 25, *s* = 3, *p* = 12) + BN + ReLU	64 × 2667
Max-pool1	MaxPool1D(*k* = 2)	64 × 1333
Conv2	64 Conv1D(*k* = 25, *s* = 3, *p* = 14) + BN + ReLU	64 × 446
Max-pool2	MaxPool1D(*k* = 2)	64 × 223
Conv3	64 Conv1D(*k* = 25, *s* = 3, *p* = 16) + BN + ReLU	64 × 78
Max-pool3	MaxPool1D(*k* = 3)	64 × 26
LSTM	64 Bi-LSTM	128 × 26
Linear	FC(*out_dim* = 2)	2
(**b**) The structure of 2D CRNN, which inputs the mel-spectogram image		
**Layer Name**	**Composition**	**Output Size**
Input	-	41 × 41
Conv1	64 Conv1D(*k* = 5, *s* = 2, *p* = 2) + BN + ReLU	64 × 21
Conv2	64 Conv1D(*k* = 5, *s* = 2, *p* = 2) + BN + ReLU	64 × 11
Conv3	64 Conv1D(*k* = 5, *s* = 2, *p* = 2) + BN + ReLU	64 × 6
LSTM	64 Bi-LSTM	128 × 6
Linear	FC(*out_dim* = 2)	2

**Table 5:** The performance of the various mobile-sized deep learning models on WSED and VCAD tasks. CNN [17], CRNN [18], and Energy Filter [5] were utilized for the baseline models. For all models except the Energy Filter, we calculated the 5-times averaged accuracy on each task. Bold text means highest score.

Task	Model	Representation	Accuracy (%)
WSED	CNN	Signal (1D)	93.01 ± 0.34
	CRNN	Signal (1D)	92.42 ± 0.23
	CNN	Mel-spectrogram (2D)	**96.02 ± 0.08**
	CRNN	Mel-spectrogram (2D)	95.51 ± 0.07
VCAD	Energy Filter	Signal (1D)	64.86
	CNN	Signal (1D)	92.40 ± 0.63
	CRNN	Signal (1D)	93.63 ± 0.32
	CNN	Mel-spectrogram (2D)	95.67 ± 0.29
	CRNN	Mel-spectrogram (2D)	**96.37 ± 0.68**

**Table 6:** Performance of mobile-sized deep learning models on multi-class voice and weapon sound event detection. CNN [17] and CRNN [18] were utilized as baseline models. The accuracy is calculated as the average over 5 runs. Bold text means highest score.

Model	Representation	Accuracy (%)
CNN	Signal (1D)	90.45 ± 0.33
CRNN	Signal (1D)	88.78 ± 0.41
CNN	Mel-spectrogram (2D)	**94.25 ± 0.16**
CRNN	Mel-spectrogram (2D)	92.28 ± 0.15

**Table 7:** WSED and VCAD performance of our 2D CNN model when the label resolution is downsampled to 2, 4, and 8 s. Bold text means highest score.

Task	Resolution (s)	Accuracy (%)
WSED	0.5	**96.02 ± 0.08**
	2.0	93.58 ± 0.19
	4.0	88.85 ± 0.82
	8.0	75.06 ± 3.69
VCAD	0.5	**95.67 ± 0.29**
	2.0	83.34 ± 0.52
	4.0	75.24 ± 1.12
	8.0	72.17 ± 1.08

## Data Availability

The data presented in this study are openly available at https://sites.google.com/view/battlesound (accessed on 5 January 2023).
